# Sulfur Amino Acid Restriction Mitigates High-Fat Diet-Induced Molecular Alterations in Cardiac Remodeling Primarily via FGF21-Independent Mechanisms

**DOI:** 10.3390/nu16244347

**Published:** 2024-12-17

**Authors:** Filipe Pinheiro, Hannah Lail, João Sérgio Neves, Rita Negrão, Desiree Wanders

**Affiliations:** 1 Department of Nutrition, Georgia State University, 140 Decatur St SE, Atlanta, GA 30303, USA; up201702955@up.pt (F.P.); hland2@student.gsu.edu (H.L.); 2 Unit of Biochemistry, Department of Biomedicine, Faculty of Medicine, University of Porto, 4200-319 Porto, Portugal; ritabsn@med.up.pt; 3 Center for Health Technology and Services Research—CINTESIS@RISE, Faculty of Medicine, University of Porto, 4200-319 Porto, Portugal; 4 Department of Chemistry, Georgia State University, 100 Piedmont Ave., Atlanta, GA 30303, USA; 5 Department of Endocrinology, Diabetes and Metabolism, Centro Hospitalar Universitário de São João, Alameda Hernâni Monteiro, 4200-319 Porto, Portugal; jsneves@med.up.pt; 6 Unit of Cardiovascular Research and Development—Unic@RISE, Department of Surgery and Physiology, Faculty of Medicine, University of Porto, Alameda Hernâni Monteiro, 4200-319 Porto, Portugal

**Keywords:** sulfur amino acid restriction, methionine restriction, high-fat diet, fibroblast growth factor 21, cardiac remodeling, metabolic health

## Abstract

**Background/Objectives:** Dietary sulfur amino acid restriction (SAAR) elicits various health benefits, some mediated by fibroblast growth factor 21 (FGF21). However, research on SAAR’s effects on the heart is limited and presents mixed findings. This study aimed to evaluate SAAR-induced molecular alterations associated with cardiac remodeling and their dependence on FGF21. **Methods:** Male C57BL/6J wild-type and FGF21 knockout mice were randomized into four dietary regimens, including normal fat and high-fat diets (HFDs) with and without SAAR, over five weeks. **Results:** SAAR significantly reduced body weight and visceral adiposity while increasing serum FGF21 levels. In the heart, SAAR-induced molecular metabolic alterations are indicative of enhanced lipid utilization, glucose uptake, and mitochondrial biogenesis. SAAR also elicited opposing effects on the cardiac gene expression of FGF21 and adiponectin. Regarding cellular stress responses, SAAR mitigated the HFD-induced increase in the cardiac expression of genes involved in oxidative stress, inflammation, and apoptosis, while upregulating antioxidative genes. Structurally, SAAR did not induce alterations indicative of cardiac hypertrophy and it counteracted HFD-induced fibrotic gene expression. Overall, most alterations induced by SAAR were FGF21-independent, except for those related to lipid utilization and glucose uptake. **Conclusions:** Altogether, SAAR promotes cardiac alterations indicative of physiological rather than pathological remodeling, primarily through FGF21-independent mechanisms.

## 1. Introduction

Cardiovascular disease (CVD) is the leading cause of disease burden worldwide, accounting for approximately one-third of all deaths [1,2]. Although the pathophysiological mechanisms leading to the development of CVD are complex and multifactorial, cardiac remodeling stands out as a critical component of its development and clinical presentation [3]. Cardiac remodeling refers to a complex series of molecular, cellular, and interstitial alterations within the heart, arising in response to a wide range of stimuli [4]. Considering the nature of the stimuli, cardiac remodeling can be classified as either physiological or pathological remodeling. Physiological remodeling is an adaptation of the heart on account of increased physiological requirements, such as in the case of exercise and pregnancy. This process involves balanced and coordinated alterations, including enhancements of oxidative metabolism and cell survival, which are clinically manifested as improvements in cardiac function [5,6]. In contrast, pathological remodeling is a maladaptation of the heart due to either chronic stress, as in the case of hypertension or metabolic disturbances, or following an acute injury, such as in myocardial infarction. This is associated with a series of detrimental molecular and structural alterations in the heart, including increased oxidative stress, chronic inflammation, chamber dilation, and fibrosis, which ultimately impair cardiac function. Unlike physiological remodeling, pathological remodeling is usually irreversible and is associated with the development and progression of CVD [5,6,7].

Dietary risks are the leading behavioral contributors to age-standardized CVD burden [2]. In this context, high-fat diets (HFD) are commonly used as research models to mimic the metabolic disorders that induce cardiac maladaptations in populations with excessive caloric and saturated fat intake [8,9]. Contrasting this, the restriction of proteinogenic sulfur amino acids—methionine and cysteine—through a dietary regimen known as sulfur amino acid restriction (SAAR) has been shown to markedly improve several cardiometabolic risk markers in rodent models. These outcomes include a reduction in body weight and visceral adiposity, enhancement of insulin sensitivity, and improvements in serum lipid profile [10,11,12]. While human data are limited, short-term clinical trials indicate that these SAAR benefits may translate to humans as well [13,14]. Since sulfur amino acids are naturally less prevalent in plant foods than in animal foods, this nutritional difference might play a role in the association between plant protein consumption and a reduced incidence of CVD mortality [15].

Recent studies have started to elucidate the molecular mechanisms underlying the benefits of SAAR, in particular the role of fibroblast growth factor 21 (FGF21). FGF21, a stress-response hormone with an important role in energy homeostasis [16], has been shown to be a key mediator of several SAAR-induced metabolic benefits. Specifically, FGF21 mediates the effects of SAAR on energy expenditure, insulin sensitivity, and serum triglyceride levels, as shown in loss-of-function studies in rodents [12,17]. While it is unknown whether FGF21 mediates the potential cardiac implications of SAAR, several reasons suggest it might. Firstly, key metabolic effects of SAAR evidenced to impact cardiac health—insulin sensitivity and serum triglyceride levels—are dependent on FGF21 [12]. Secondly, SAAR enhances cardiac glucose uptake in an FGF21-dependent manner [12]. Thirdly, FGF21 itself has cardioprotective properties, capable of counteracting the process of pathological cardiac remodeling. Animal models have shown that FGF21 mitigates abnormal cellular stress responses, such as oxidative stress, inflammation, and apoptosis, in the heart, while reducing cardiac hypertrophy and fibrosis. This ultimately improves cardiac function and prevents adverse cardiac outcomes [18,19]. In addition, FGF21 was shown to improve certain cardiometabolic risk factors in humans [19].

Despite numerous studies supporting the benefits of SAAR in certain cardiometabolic risk markers, research into its direct effects on the heart remains limited and presents mixed results. On one hand, SAAR was shown to improve cardiac dysfunction in middle-aged obese male mice [20] and to reduce the production of mitochondrial reactive oxygen species (ROS) within rat hearts, along with a concomitant reduction in mitochondrial DNA oxidative damage [21,22,23]. On the other hand, SAAR-induced markers are associated with detrimental effects on the heart, including hyperhomocysteinemia in both rodents [24] and humans [14], and increased heart weight-to-body weight (HW/BW) ratios, prolonged QRS intervals, and elevated levels of natriuretic peptides in both young and aged male mice [24,25]. Similarly, protein restriction, which also limits sulfur amino acid intake, elicits adverse cardiac effects in young male rodents, leading to cardiac dysfunction [26,27,28,29].

Given the limited and conflicting evidence regarding the effects of SAAR on cardiac health, particularly in young male animals, we aimed to comprehensively evaluate the effects of SAAR on cardiac remodeling in high-fat-fed young male mice. We focused on examining specific molecular markers in the heart related to metabolism, cellular stress responses, and structural integrity that are distinctly modulated by physiological and pathological cardiac remodeling. In addition, due to the relevance of FGF21 in mediating several biological responses of SAAR, we also aimed to investigate whether the cardiac effects of SAAR are dependent on FGF21. By achieving these aims, the present work seeks to advance our understanding of the interactions between SAAR, cardiac health, and FGF21, potentially guiding future therapeutic strategies for the prevention and treatment of CVD.

## 2. Materials and Methods

### 2.1. Animal Studies

All animal experiments were reviewed and approved by the Institutional Animal Care and Use Committee (IACUC) (Approval Code: A21030, Approval Date: 22 January 2021) at Georgia State University, ensuring compliance with the National Research Council guidelines, Animal Welfare Act, and Public Health Service Policy on the humane care and use of laboratory animals. Following seven days of acclimation, male C57BL/6J wild-type (WT) (Jax#000664) and global FGF21 knockout (*Fgf21^−^^/^^−^*) (Jax#033846) mice (Jackson Laboratories, Bar Harbor, ME, USA), aged five to six weeks, were randomly assigned to one of four dietary regimens (WT: 13–14 per diet; *Fgf21^−^^/^^−^*: 10–11 per diet) for five weeks: (1) normal-fat diet (NFD) (3.815 kcal/g, 14.8% of energy from protein, 64.9% of energy from carbohydrates, 18.9% of energy from fat, 0.86% methionine); (2) normal-fat diet with SAAR (NFD + SAAR) (0.17% methionine); (3) HFD (5.45 kcal/g, 14.7% of energy from protein, 25.7% of energy from carbohydrate, 59.6% of energy from fat, 0.86% methionine); (4) HFD + SAAR (0.17% methionine). All diets were formulated using a semi-purified elemental amino acid-based diet obtained from Dyets Inc. (Bethlehem, PA, USA) as extruded pellets. The total absolute content of amino acids present in the diets of both HFD groups was higher (except for methionine) to match the relative protein content present in the diets of the NFD groups (14.8% of energy from protein). Cysteine was not present in any diet. This feeding paradigm was based on previous studies [30,31]. The experimental design of the study and the nutritional composition of each diet are detailed in the Supplementary Materials. Food and water were provided ad libitum. After 5 weeks, the animals were sacrificed by decapitation following CO_2_-induced narcosis. Heart, liver, and visceral adipose tissue depots—epididymal white adipose tissue (eWAT) and retroperitoneal white adipose tissue (rpWAT)—were promptly dissected, weighed, and snap-frozen in liquid nitrogen. Trunk blood was also collected, from which serum was isolated by centrifugation at 5000 rpm for 15 min using an accuSpin Micro 17R (Fisher Scientific, Hampton, NH, USA). Tissue and serum samples were stored at −80 °C for subsequent analysis of protein and gene expression.

### 2.2. Enzyme-Linked Immunosorbent Assay (ELISA)

Fasting serum FGF21 concentrations of a subset of animals (*n* = 4–6 per group) were measured using the commercial Quantikine Mouse/Rat FGF21 ELISA Kit (Catalog#: MF2100, R&D Systems, Minneapolis, MN, USA) according to the manufacturer’s instructions. The FGF21 concentrations were determined by reading the optical density at 450 nm using a Synergy HT microplate reader (BioTek Instruments, Inc., Winooski, VT, USA).

### 2.3. Immunoblotting

Heart ventricles were lysed in radioimmunoprecipitation assay (RIPA) buffer containing protease and phosphatase inhibitors and ethylenediaminetetraacetic acid (EDTA). The lysates were then centrifuged at 16,000× *g* for 15 min at 4 °C, and the supernatant was carefully collected. Total protein concentrations were determined in the supernatant using the DC protein assay kit (Bio-Rad Laboratories, Hercules, CA, USA). A total of 50 µg of protein was subsequently combined with 4x Laemmli buffer and 10% 2-mercaptoethanol, briefly mixed and centrifuged, and then denatured by heating to 70 °C for 10 min on a dry heating block. The denatured proteins were separated using 12% SDS-PAGE gels and blotted onto PVDF membranes (ThermoFisher Scientific, Waltham, MA, USA) using the Trans-Blot Turbo transfer system (Bio-Rad Laboratories). The membranes were then blocked in 5% non-fat dry milk (NFDM) and washed in TBS-T (3× for 5 min) at room temperature, followed by incubation with primary antibodies, made up in 5% BSA (1:1000 dilution), for 16 h at 4 °C. After incubation, the membranes were washed in TBS-T (3× for 5 min) and then incubated with anti-mouse secondary antibodies (#515-035-003—Jackson Laboratories) diluted in 5% NFDM to 1:5000, for 1 h at room temperature, and then washed again in TBS-T (3× for 5 min). Protein bands were detected by chemiluminescence, using HRP (Immobilon Forte Western HRP Substrate, Millipore, Burlington, MA, USA), and images were obtained with the ChemiDoc Imaging System (Bio-Rad Laboratories). Immunoblots were performed using primary antibodies against COXIV (#ab14744—Abcam, Cambridge, UK). Protein was quantified by normalizing protein band density to total lane protein using Image Lab 6.0 software from Bio-Rad Laboratories.

### 2.4. Gene Expression

RNA was extracted and isolated from heart ventricles using the Qiazol Lysis Reagent (Qiagen, Germantown, MD, USA) according to the manufacturer’s instructions. After extraction, the concentration and purity of the RNA were determined using the Nanodrop ND 1000 spectrophotometer (Nanodrop Technologies, Wilmington, DE, USA). RNA sample purity ranged from 1.8 to 2.0 for the E_260_/E_280_ ratio and from 1.9 and 2.2 for the E_260_/E_230_ ratio. A total of 2 µg of RNA was used to synthesize cDNA using a reverse transcription system (Promega, Madison, WI, USA) in a C-1000 Touch Thermal Cycler (Bio-Rad Laboratories). The generated cDNA was aliquoted and stored at −80 °C for use. Gene expression was measured by real-time PCR using a LightCycler 96 (Roche, Penzberg, Germany) with SYBR Green (MedChemExpress, South Brunswick, NJ, USA) as the detection method. Targeted genes are listed in the Supplementary Materials. The relative quantification of gene expression was determined through the 2^ΔΔCT^ method [32], with *Ppia* and *Gapdh* mRNA expressions serving as housekeeping controls.

### 2.5. Data Analysis

All results were normalized to the NFD group of the respective genotypes. Outliers were identified and excluded using the ROUT method with a 1% false discovery rate. Data were analyzed using one-way ANOVA to evaluate differences between means among the multiple dietary groups within each genotype. In addition, Tukey–Kramer post-hoc tests were applied to account for multiple comparisons in the identification of differences between specific groups. Simple linear regressions were performed to examine the linear relationships between serum FGF21 levels and cardiac *Fgf21* and liver *Fgf21* mRNA expressions. Furthermore, as it is recommended to use ANCOVA to account for a potential confounding effect of body weight when comparing relative organ weights, as not controlling for such confounding can lead to incorrect conclusions [33], an ANCOVA test was performed with HW/BW as the dependent variable, body weight as the covariate, and dietary group as the independent factor. All data are presented as the mean ± standard error of the mean (SEM). Differences were deemed significant if *p* ≤ 0.05. GraphPad Prism 9.0 (GraphPad Software, San Diego, CA, USA) was used for all statistical analysis, except for ANCOVA, which was performed using IBM SPSS Statistics version 29 (IBM Corp., North Castle, NY, USA).

## 3. Results

### 3.1. Body Weight, Visceral Adiposity, and Cardioprotective Hormones

#### 3.1.1. SAAR Reduced Body Weight and Visceral Fat Independently of FGF21

**Figure 1:**
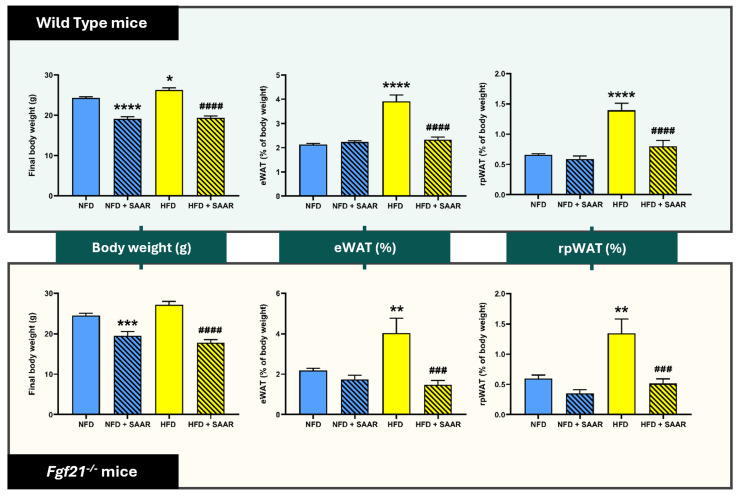
SAAR reduced body weight and visceral adiposity independently of FGF21. Body weight (*n* = 10–14 animals per group) was measured prior to sacrifice. The weights of eWAT and rpWAT were measured post-sacrifice (*n* = 7–11 animals per group). Statistical analysis was performed using one-way ANOVA followed by Tukey–Kramer post-hoc multiple comparison tests. Significance levels to the NFD group are denoted by the * symbol: * *p* < 0.05, ** *p* < 0.01, *** *p* < 0.001, **** *p* < 0.0001. Significance levels to the HFD group are denoted by the ^#^ symbol: ^###^ *p* < 0.001, and ^####^ *p* < 0.0001. Abbreviations: eWAT, epididymal white adipose tissue; rpWAT, retroperitoneal white adipose tissue.

The final body weight and the weight percentages of visceral adipose tissue depots (eWAT and rpWAT) are presented in Figure 1. SAAR-fed mice showed a significant reduction in body weight compared to their respective controls (NFD and HFD groups) after 5 weeks of intervention, in both WT and *Fgf21^−^^/^^−^* mice. HFD significantly increased body weight in WT, but not in *Fgf21^−^^/^^−^* mice. Regarding visceral adiposity, SAAR counteracted the HFD-induced increase in eWAT and rpWAT in both genotypes.

#### 3.1.2. SAAR Modulated the Expression Levels of the Cardioprotective Hormones FGF21 and Adiponectin

**Figure 2:**
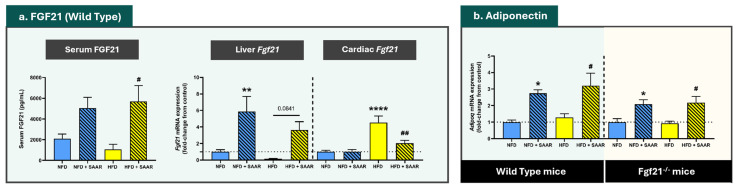
SAAR modulated the expression levels of the cardioprotective hormones FGF21 and Adiponectin. Serum FGF21 levels were quantified using an ELISA kit (*n* = 4–6 animals per group). mRNA expression levels were quantified via RT-PCR (*n* = 7–11 animals per group). Statistical analysis was performed using one-way ANOVA followed by Tukey–Kramer post-hoc multiple comparison tests. Significance levels to the NFD group are denoted by the * symbol: * *p* < 0.05, ** *p* < 0.01, **** *p* < 0.0001. Significance levels to the HFD group are denoted by the ^#^ symbol: ^#^ *p* < 0.05 and ^##^ *p* < 0.01.

**Figure 3:**
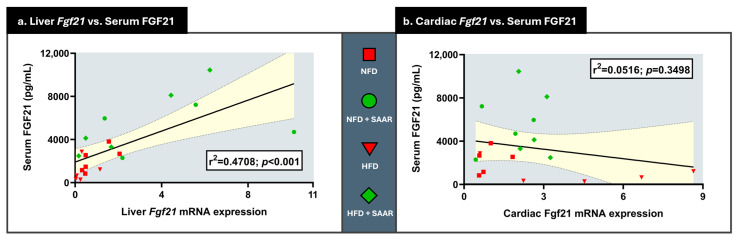
Serum FGF21 levels are associated with Liver *Fgf21* mRNA expression but not with Cardiac *Fgf21* mRNA expression. Serum FGF21 levels were quantified using an ELISA kit. mRNA expression levels were quantified via RT-PCR. Relationship between serum FGF21 concentrations and liver Fgf21 (*n* = 20) and cardiac Fgf21 (*n* = 19) mRNA concentrations was determined with linear regression analysis. The errors of the linear regression lines are denoted in yellow.

Compared to NFD, exposure to HFD did not change serum FGF21 levels or liver *Fgf21* mRNA expression in WT animals (Figure 2a). In contrast, SAAR elevated both serum FGF21 concentrations and liver *Fgf21* mRNA expression, while abrogating the HFD-induced increase in cardiac *Fgf21* mRNA expression (Figure 2a). A significant positive relationship (r^2^ = 0.471; *p* < 0.001) was identified between liver *Fgf21* mRNA expression and serum FGF21 levels (Figure 3a), suggesting that liver *Fgf21* mRNA expression levels are associated with higher serum FGF21 concentrations. In contrast, there was no significant relationship between cardiac *Fgf21* mRNA expression and serum FGF21 concentrations (r^2^ = 0.052; *p* = 0.3498) (Figure 3b). Regarding adiponectin expression, SAAR increased cardiac *Adipoq* mRNA expression in a genotype-independent manner (Figure 2b).

### 3.2. Cardiac Metabolism

#### 3.2.1. SAAR Increased the Expression of Lipid Utilization Genes and Slc2a1 (GLUT1) in WT Mice but Not in *Fgf21^−^^/^^−^* Mice

**Figure 4:**
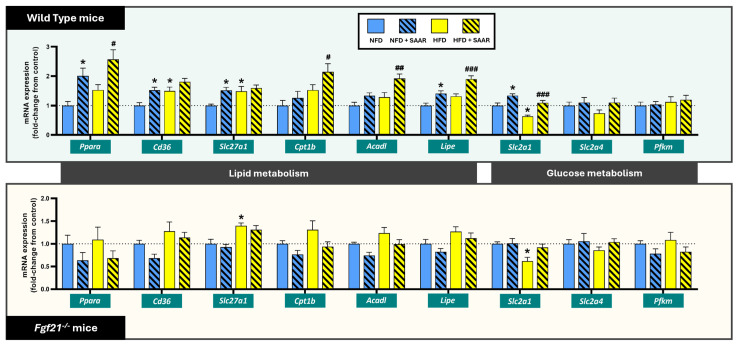
SAAR enhanced the expression of cardiac lipid utilization genes and *Slc2a1* in WT mice but not in *Fgf21^−^^/^^−^* mice. mRNA expression levels were quantified via RT-PCR (*n* = 6–11 animals per group). Statistical analysis was performed using one-way ANOVA followed by Tukey–Kramer post-hoc multiple comparison tests. Significance levels to the NFD group are denoted by the * symbol: * *p* < 0.05. Significance levels to the HFD group are denoted by the ^#^ symbol: ^#^ *p* < 0.05, ^##^ *p* < 0.01 and ^###^ *p* < 0.001.

SAAR enhanced the cardiac expression of several genes involved in peroxisome proliferator-activated receptor alpha (PPARα) signaling in WT mice, but not in *Fgf21^−^^/^^−^* mice (Figure 4). Specifically, SAAR upregulated the mRNA expression levels of *Ppara* and several PPARα-target genes, including genes involved in fatty acid uptake (i.e., *Cd36* and *Slc27a1*) relative to the NFD group, mitochondrial β-oxidation (i.e., *Cpt1b* and *Acadl*) relative to the HFD group, and triglyceride turnover (i.e., *Lipe*) in comparison to both controls. The expression of *Gpam*, a PPARα-target gene involved in triglyceride turnover, remained unchanged by SAAR (Supplementary Materials). In contrast, HFD did not significantly alter the mRNA expression levels of *Ppara* and most of the PPARα-target genes (i.e., *Cpt1b*, *Acadl*, and *Lipe*), other than the upregulation of the genes involved in fatty acid uptake: *Slc27a1*, across both genotypes, and *Cd36*, in WT mice only (Figure 4). Regarding glucose utilization, SAAR significantly increased the mRNA expression of the gene encoding for the glucose transporter, GLUT1 (*Slc2a1*), in WT but not *Fgf21^−^^/^^−^* mice, whereas HFD led to an opposite result across both genotypes (Figure 4). The mRNA expression levels of other glucose utilization genes, specifically genes encoding for the glucose transporter GLUT4 (*Slc2a4*) and the glycolytic enzyme PFKM (*Pfkm*), remained unaffected either by SAAR or HFD across genotypes (Figure 4).

#### 3.2.2. SAAR Increased the Expression of Genes Related to Mitochondrial Biogenesis Across Both Genotypes

**Figure 5:**
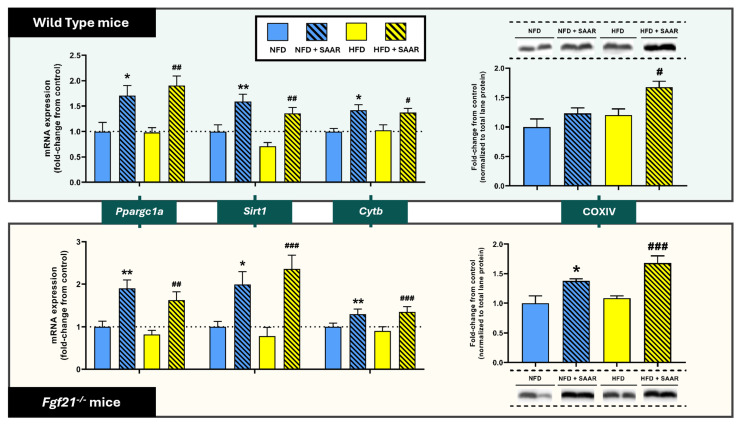
SAAR modulated the expression of genes related to mitochondrial biogenesis across both genotypes in the heart. mRNA expression levels were quantified via RT-PCR (*n* = 7–11 animals per group). Protein quantification was conducted using Western blot (*n* = 6–11 animals per group). Statistical analysis was performed using one-way ANOVA followed by Tukey–Kramer post-hoc multiple comparison tests. Significance levels to the NFD group are denoted by the * symbol: * *p* < 0.05 and ** *p* < 0.01. Significance levels to the HFD group are denoted by the ^#^ symbol: ^#^ *p* < 0.05, ^##^ *p* < 0.01 and ^###^ *p* < 0.001.

SAAR increased the transcription of genes involved in mitochondrial biogenesis, specifically *Ppargc1a* and *Sirt1*, in both WT mice and *Fgf21^−^^/^^−^* mice (Figure 5), while not altering the expression levels of *Tfam* (Supplementary Materials). Consistent with this, SAAR led to an increase in the expression of enzymes involved in the mitochondrial electron transport chain (ETC) across both genotypes, specifically the protein expression of COXIV and mRNA expression of *Cytb*, the gene encoding cytochrome b, a subunit of complex III (Figure 5).

### 3.3. Cardiac Cellular Stress Responses

#### 3.3.1. SAAR Modulated the Oxidative Stress Response by Increasing the Expression of Antioxidant Enzymes and Abrogating HFD-Induced Increases in Nox2 in Both Genotypes

**Figure 6:**
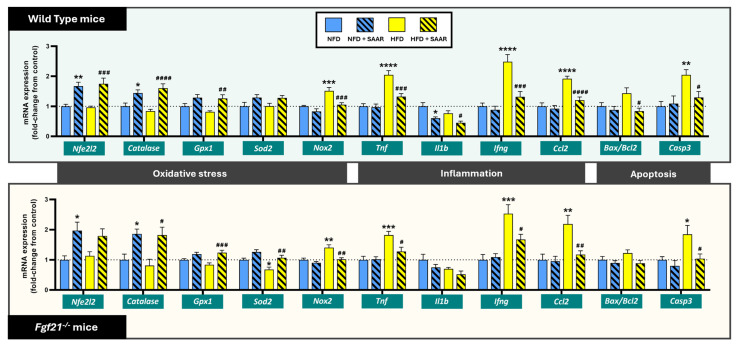
SAAR abrogated the HFD-induced expression of genes associated with abnormal cellular stress responses across both genotypes in the heart. mRNA expression levels were quantified via RT-PCR (*n* = 6–11 animals per group). Statistical analysis was performed using one-way ANOVA followed by Tukey–Kramer post-hoc multiple comparison tests. Significance levels to the NFD group are denoted by the * symbol: * *p* < 0.05, ** *p* < 0.01, *** *p* < 0.001, **** *p* < 0.0001. Significance levels to the HFD group are denoted by the ^#^ symbol: ^#^ *p* < 0.05, ^##^ *p* < 0.01, ^###^ *p* < 0.001, and ^####^ *p* < 0.0001.

SAAR significantly increased the gene expression of *Nfe2l2*, a key transcription factor involved in the regulation of the antioxidant response, and the antioxidant enzymes *Cat* and *Gpx1* in both WT and *Fgf21^−^^/^^−^* mice (Figure 6). In addition, SAAR significantly abrogated the HFD-induced decrease in mRNA expression of the antioxidant enzyme *Sod2* in *Fgf21^−^^/^^−^* mice only. In contrast, SAAR abrogated the HFD-induced increase in *Nox2* mRNA expression, involved in the production of ROS, across both genotypes (Figure 6).

#### 3.3.2. SAAR Decreased Inflammatory Gene Expression Independently of FGF21

SAAR mitigated the HFD-induced mRNA expression of the inflammatory cytokines *Tnf* and *Ifng*, as well as the chemokine *Ccl2* across both genotypes (Figure 6). Furthermore, SAAR significantly decreased the mRNA expression of the inflammatory cytokine *Il1b* irrespective of diet, but only in WT mice. In contrast, HFD did not alter *Il1b* expression in either genotype (Figure 6).

#### 3.3.3. SAAR Decreased Apoptotic mRNA Expression in WT and *Fgf21^−/−^* Mice

SAAR mitigated the HFD-induced increase in mRNA expression of the pro-apoptotic marker *Casp3* across both genotypes (Figure 6). Furthermore, the pro-apoptotic marker *Bax*/*Bcl2* ratio was significantly reduced by SAAR in relation to the HFD group in WT mice only (Figure 6). In contrast, the individual gene expression of the pro-apoptotic genes *Bax* and *Casp9*, and of the anti-apoptotic gene *Bcl2*, was not significantly altered by SAAR (Supplementary Materials).

### 3.4. Cardiac Structural Integrity

#### 3.4.1. SAAR Enhanced HW/BW Ratios and Natriuretic Peptide Expression Without Affecting the Expression of Other Hypertrophic Fetal Genes

**Figure 7:**
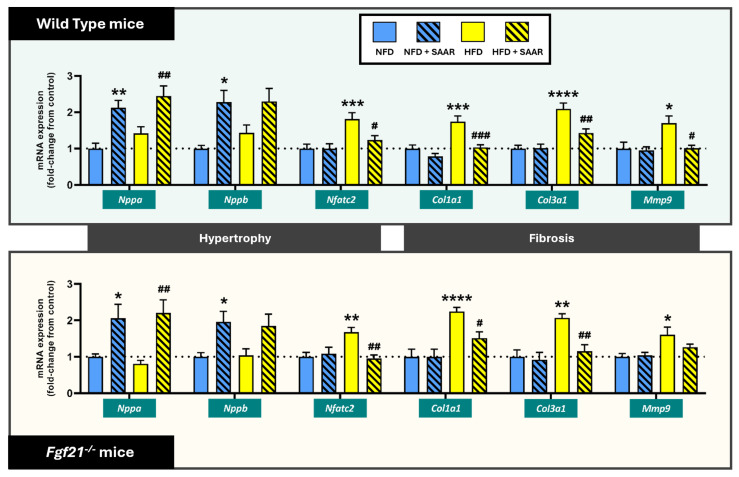
SAAR modulated the expression of genes associated with cardiac hypertrophy and fibrosis pathways in both genotypes. mRNA expression levels were quantified via RT-PCR (*n* = 7–11 animals per group). Statistical analysis was performed using one-way ANOVA followed by Tukey–Kramer post-hoc multiple comparison tests. Significance levels to the NFD group are denoted by the * symbol: * *p* < 0.05, ** *p* < 0.01, *** *p* < 0.001, **** *p* < 0.0001. Significance levels to the HFD group are denoted by the ^#^ symbol: ^#^ *p* < 0.05, ^##^ *p* < 0.01 and ^###^ *p* < 0.001.

Although mice displayed similar heart weights after the different dietary regimens, SAAR increased HW/BW ratios in both WT mice (F (3,36) = 4.37, *p* = 0.01) and *Fgf21^−^^/^^−^* mice (F (3,26) = 6.32, *p* = 0.002), according to one-way ANOVA results (Table 1). However, the differences in HW/BW ratios were no longer significant after adjusting for body weight using ANCOVA analysis, in both WT mice (F (3, 35) = 0.63, *p* = 0.691) and *Fgf21^−^^/^^−^* mice (F (3, 35) = 1.35, *p* = 0.28). Moreover, body weight was identified as a significant covariate in WT mice (F (1, 35) = 6.274, *p* = 0.001), but not in *Fgf21^−^^/^^−^* mice (F (1, 35) = 12.56, *p* = 0.231). These results suggest that the differences in HW/BW ratios observed after one-way ANOVA analysis are confounded by variations in body weight. Regarding gene expression studies, SAAR increased the cardiac mRNA expression levels of the natriuretic peptides *Nppa* and *Nppb*, regardless of genotype (Figure 7). Despite this, SAAR did not affect the mRNA expression of other genes associated with the hypertrophic fetal gene program such as *Srf*, *Gata4*, *Gata6*, *Myh7*, *Myh6,* or the *Myh7/Myh6* ratio (Supplementary Materials), and even reduced the HFD-induced expression of *Nfatc2*, across both genotypes (Figure 7).

#### 3.4.2. SAAR Abrogated HFD-Induced Fibrotic mRNA Expression Across Both Genotypes

SAAR counteracted the HFD-induced upregulation of fibrotic-related genes, specifically the mRNA expression of the primary cardiac collagen isoforms *Col1a1* and *Col3a1*, as well as of the metalloproteinase *Mmp9*, in both WT and *Fgf21^−^^/^^−^* mice (Figure 7). However, the reduction in *Mmp9* expression by SAAR in *Fgf21^−^^/^^−^* mice did not reach statistical significance (*p* = 0.264). In contrast, the mRNA expression of *Mmp2* was not significantly altered by either HFD or SAAR dietary regimens (Supplementary Materials).

## 4. Discussion

**Figure 8:**
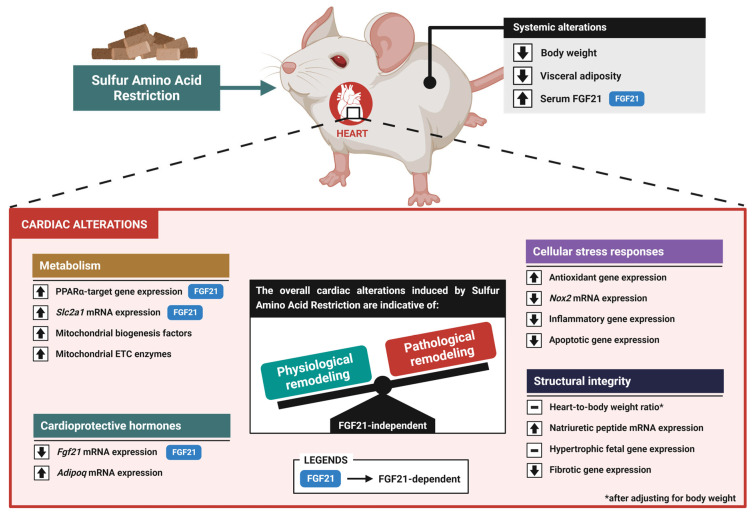
SAAR-induced molecular alterations in the heart are more indicative of physiological than pathological cardiac remodeling. Created with BioRender.com.

Previous studies have demonstrated that SAAR improves various cardiometabolic risk markers, implicating FGF21 as a critical mediator for some of these processes [12,17]. Despite these observations, research into the direct implications of SAAR in the heart is limited, presenting a somewhat contradictory landscape. This study revealed that, systemically, SAAR abrogated the HFD-induced increase in body weight and visceral adiposity while increasing serum FGF21 levels. Furthermore, SAAR elicited several outcomes in the heart: (1) induced alterations consistent with an enhanced myocardial energy metabolism; (2) differentially modulated the mRNA expression of the cardioprotective hormones FGF21 and adiponectin; (3) counteracted the HFD-induced expression of genes involved in cellular stress responses; (4) increased HW/BW ratios and natriuretic peptide gene expression—markers of cardiac hypertrophy. However, HW/BW ratio differences were confounded by body weight, and the expression of other cardiac hypertrophic genes was unaltered; and (5) abrogated the HFD-induced upregulation of genes associated with cardiac fibrosis. All these alterations, except for the molecular alterations related to lipid utilization and glucose uptake, were also observed in *Fgf21^−^^/^^−^* mice. Altogether, SAAR induces alterations that align more with physiological rather than pathological cardiac remodeling, primarily through FGF21-independent mechanisms (Figure 8).

The accumulation of visceral adiposity is a hallmark outcome of an HFD, inducing a systemic cascade of metabolic disturbances, such as hyperlipidemia and chronic inflammation, with deleterious effects on cardiac health [8,34]. Consistent with previous reports [12,35], SAAR counteracts this cardiometabolic risk factor by decreasing body weight and visceral adiposity, potentially alleviating systemic stress to the heart. Furthermore, although the heart obtains most of its energy from the mitochondrial β-oxidation of fatty acids [36,37], an oversupply of fatty acids from an HFD can quickly disrupt its normal metabolic balance [38], even without a significant accumulation of body mass [39,40]. This is characterized by a metabolic inefficient shift towards cardiac fatty acid oxidation at the expense of glucose utilization, accompanied by impaired mitochondrial dynamics, which ultimately leads to abnormal cellular stress responses and structural maladaptations [34,41,42,43]. This phenotype is observed in certain pathological states, including diabetic cardiomyopathy [34,44]. Within this metabolic framework, by increasing the mRNA expression levels of *Ppara*—a nuclear receptor with a crucial role in myocardial lipid utilization [45,46]—and its target genes, SAAR seems to increase the capacity of fatty acid utilization in the heart. This, along with the increased expression of the glucose transporter *Slc2a1* and the unchanged expression of other genes involved in glucose metabolism (*Slc2a4* and *Pfkm*), suggests that SAAR increases lipid utilization without compromising the heart’s overall capacity for glucose utilization, at least at the transcriptional level. Further supporting these findings are the observed increases in the expression of mitochondrial ETC enzymes (*Cytb* and COXIV) and biogenesis factors (*Ppargc1a* and *Sirt1*), indicating SAAR enhances mitochondrial biogenesis. Altogether, the metabolic alterations induced by SAAR are similar to the increased myocardial energy metabolism observed in certain physiological states [6,44,47,48], suggesting beneficial cardiac remodeling (Figure 8).

The differential response induced by SAAR with regard to FGF21 and adiponectin in the heart reveals a tissue-specific regulation of these cardioprotective hormones. The increase in serum FGF21 levels following SAAR is consistent with previous reports [49], as FGF21 is primarily induced by a transcriptional program in the liver in response to this metabolic challenge [50]. However, unlike the liver, the heart is not considered a major site of FGF21 production, contributing minimally to serum FGF21 levels [16,51]. Congruent to this, we found that serum FGF21 levels were correlated with liver *Fgf21* expression, but not with cardiac *Fgf21* expression. The abrogation of HFD-induced cardiac *Fgf21* expression in the context of SAAR, however, suggests a heart-specific regulation mechanism. While increased serum levels of FGF21 are generally associated with physiological states such as veganism [52] or exercise [53], cardiac *Fgf21* expression is described to be enhanced by pathological stimuli, such as diabetic cardiomyopathy and heart failure [16]. As a result, local cardiac *Fgf21* levels may reflect the heart’s stress adaptation to HFD-induced stress, with SAAR mitigating this stress. Further supporting these findings, SAAR increased the transcriptional expression of cardiac adiponectin, an adipokine with numerous cardioprotective effects [54].

An HFD induces a series of cellular stress responses in the heart, resulting in significant increases in oxidative stress, inflammation, and apoptosis, all of which are reflected at the transcriptional level [34,55,56]. In this study, we observed a transcriptional network of adaptive mechanisms induced by SAAR that appear to counteract the detrimental alterations caused by an HFD on these cellular stress responses. Regarding oxidative stress, SAAR abrogated the HFD-induced gene expression of *Nox2*, an important contributor to oxidative stress in cardiomyocytes [57]. Furthermore, SAAR induced the mRNA expression of genes associated with enhanced antioxidant defense, including the transcription factor *Nfe2l2*, the transcriptional co-activator *Ppargc1a,* and the deacetylase *Sirt1*, along with the antioxidant enzymes *Gpx1* and *Catalase* [58,59,60]. This coordinated transcriptional regulation of prooxidant and antioxidant enzymes suggests an enhanced antioxidative defense within the heart. Regarding inflammation, by counteracting HFD-induced mRNA expression of the pro-inflammatory cytokine *Tnf* and the chemokine *Ccl2*, SAAR counteracted HFD-induced inflammation at the transcriptional level. Additionally, SAAR decreased the expression of the cytokine *Il1b* compared to controls, further supporting its role in reducing inflammation. These findings align with previous studies reporting that SAAR reduces inflammation in other tissues [31,61]. Finally, the observed transcriptional modulation of pro-apoptotic markers, particularly the abrogation of the HFD-induced gene expression of *Casp3* and *Bax*/*Bcl2* ratio, points towards an important regulation by SAAR that mitigates excessive cell death [6,56,62]. In essence, the transcriptional alterations elicited by SAAR regarding cellular stress responses are indicative of a protective response that mitigates HFD-induced stress and damage within the cardiac tissue (Figure 8).

The alterations elicited by SAAR regarding cardiac structural markers are associated with both physiological and pathological cardiac remodeling. Notably, consistent with previous reports [24,25], SAAR increased HW/BW ratios and natriuretic peptide mRNA expression, which are normally associated with pathological cardiac hypertrophy [6]. However, several nuances should be considered while interpreting these results. Although increased HW/BW ratio is considered a marker of cardiac hypertrophy, its validity can be compromised in comparisons between groups with significant differences in body weight, as the heart does not reduce its weight in proportion to the loss of body weight [63]. As such, in SAAR-fed mice, a lower body mass may lead to an increased HW/BW ratio due to relative preservation of cardiac mass rather than reflecting cardiac hypertrophy. This interpretation is supported by the fact that body weight was identified as a significant confounding factor in the HW/BW ratio differences between dietary groups. Regarding natriuretic peptides, although their elevation typically indicates myocardial strain [64], they can also be upregulated by physiological stimuli, such as in response to exercise [65,66]. This dual role highlights the complexity of interpreting these markers. However, given the unchanged or even reduced expression of genes involved in the cardiac hypertrophic fetal gene program—major molecular hallmarks of pathological cardiac hypertrophy [6,67]—the observed increase in natriuretic peptides under SAAR likely reflects an adaptive response to altered metabolic demands rather than a result of pathological myocardial strain. Further supporting this, the observed reduction in HFD-induced fibrotic gene expression (*Col1a1*, *Col3a1*, *Mmp9*) by SAAR suggests a protective effect against the abnormal remodeling of the extracellular matrix that normally accompanies pathological cardiac hypertrophy [42,68]. Altogether, the overall alterations related to the structural integrity of the heart suggest an adaptive response to this dietary restriction, rather than pathological growth (Figure 8).

Most of the cardiac alterations previously outlined to be induced by SAAR were also conserved in *Fgf21^−^^/^^−^* mice, suggesting that these effects might not be mediated by FGF21. However, this observation is complicated by the different mechanisms through which SAAR induces the loss of body weight and visceral fat: in WT mice, it occurs via increased energy expenditure in an FGF21-dependent manner [12,17,69,70], whereas in *Fgf21^−^^/^^−^* mice, it occurs through reduced energy intake [12]. Since it is possible that the alterations of SAAR in the heart are secondary to its improvements in body composition, we cannot exclude the possibility that, in WT mice, these effects of SAAR in the heart are indirectly mediated by FGF21. As a result, additional research is needed to delineate the specific mechanisms through which SAAR exerts its cardioprotective effects. Despite this, the transcriptional upregulation of *Ppara*, along with its target genes, and *Slc2a1* was found to be FGF21-dependent. This dependency is supported by FGF21’s role in regulating lipid metabolism and energy homeostasis in the heart, where it acts as a key hormonal signal in the adaptive response to nutritional and metabolic stress [16,19]. Although there are no studies demonstrating that FGF21 directly induces cardiac PPARα expression, FGF21 is known to interact with PPARα in other tissues, such as the liver and adipose tissue, where it promotes the oxidation of fatty acids and improves overall myocardial energy metabolism [16]. Regarding the FGF21-dependent transcriptional effects on glucose uptake, a previous report demonstrated that SAAR increases cardiac glucose uptake in an FGF21-dependent manner [12]. Additionally, FGF21’s role in upregulating *Slc2a1* transcription in cardiomyocytes has also been documented [71]. Altogether, these findings underscore FGF21’s essential role in specifically mediating the effects of SAAR on lipid and glucose metabolism, suggesting that there are alternative FGF21-independent mechanisms likely involved in mediating the additional benefits of SAAR.

In summary, our findings bridge the gap between conflicting findings in previous reports regarding the complex effects of SAAR on cardiac health. However, since molecular alterations do not always translate to changes in function, future experiments are needed to validate these results at a functional level and to assess potential sex differences. Furthermore, the direct effects of FGF21 in the heart within the context of SAAR still need to be fully elucidated, considering the potential confounding effects of the different mechanisms underlying body composition alterations in *Fgf21^−^^/^^−^* mice and the systemic alterations introduced by the usage of a global FGF21 knockout model. Finally, we also acknowledge the limitations of using an animal model for this study, which may limit the direct translatability of these findings to humans. However, short-term controlled feeding studies using amino acid mixtures have shown promising results regarding the translatability of the metabolic benefits of SAAR in humans [13,14]. Nonetheless, further research is warranted to evaluate its long-term safety, feasibility, and effectiveness in disease prevention in humans. Due to the complex nature of foods, in real-life settings, it is not possible to uniquely target the dietary intake of sulfur amino acids. Therefore, implementing SAAR would likely involve adopting a plant-based dietary pattern (e.g., vegan or vegetarian diets) in light of the fact that plant-based foods have a naturally lower content of these amino acids. However, it is unclear whether plant-based diets can achieve the degree of restriction that is necessary to replicate the benefits of SAAR in humans.

## 5. Conclusions

SAAR induces a series of molecular modifications characteristic of physiological cardiac remodeling, primarily via an FGF21-independent manner. Specifically, SAAR promoted changes that suggest an overall increase in energy metabolism while protecting against HFD-induced transcriptional alterations associated with oxidative stress, inflammation, apoptosis, and fibrosis in the heart. Most of these alterations were preserved in *Fgf21^−^^/^^−^* mice, except for the transcriptional alterations on fatty acid utilization and glucose uptake, indicating that most of the benefits elicited by SAAR in the heart are not dependent on FGF21. These results are consistent with previous findings in other tissues, where SAAR improves overall metabolic health and mitigates HFD-induced cellular stress responses.

## Figures and Tables

**Table 1:** Heart weight and heart-to-body weight (HW/BW) ratio.

Variable	Genotype	Dietary Regimen			
		NFD	NFD + SAAR	HFD	HFD + SAAR
Heart Weight (mg)	WT	160.1 ± 5.55	159.9 ± 7.44	174.5 ± 9.78	154.4 ± 6.53
	*Fgf21^−^^/^^−^*	148.6 ± 7.82	148.3 ± 13.5	159.9 ± 6.69	125.8 ± 6.24
HW/BW ratio (mg/g)	WT	6.63 ± 0.21	8.12 ± 0.54 *	6.64 ± 0.31	7.87 ± 0.33
	*Fgf21^−^^/^^−^*	6.21 ± 0.25	7.77 ± 0.35 *	6.51 ± 0.40	7.73 ± 0.31

Statistical analysis was performed using one-way ANOVA followed by Tukey–Kramer post-hoc multiple comparison tests. Significance levels to the NFD group are denoted by the * symbol: * *p* < 0.05.

## Data Availability

The data presented in this study are available upon request from the corresponding author.

## Supplementary Materials

The following supporting information can be downloaded at: https://www.mdpi.com/article/10.3390/nu16244347/s1, Figure S1: Experimental design of the study; Figure S2: Expression of other genes in the heart; Table S1: Nutritional composition of each dietary regimen; Table S2: List of targeted genes in RT-PCR.
