# Supplementary material for: Sulfur Amino Acid Restriction Mitigates High-Fat Diet-Induced Molecular Alterations in Cardiac Remodeling Primarily via FGF21-Independent Mechanisms
Source: Nutrients. 2024 Dec 17;16(24):4347. doi: 10.3390/nu16244347 (PMC11677450; doi:10.3390/nu16244347)
Supplement: Supplementary file 1 [file nutrients-16-04347-s001.zip › nutrients-3346386-supplementary.pdf]

SUPPLEMENTARY MATERIAL

Figures

Supplementary Figure S1. Experimental design of the study

Supplementary Figure S2. Expression of other genes in the heart

Tables

Supplementary Table S1. Nutritional composition of each dietary regimen

Supplementary Table S2. List of targeted genes in RT-PCR

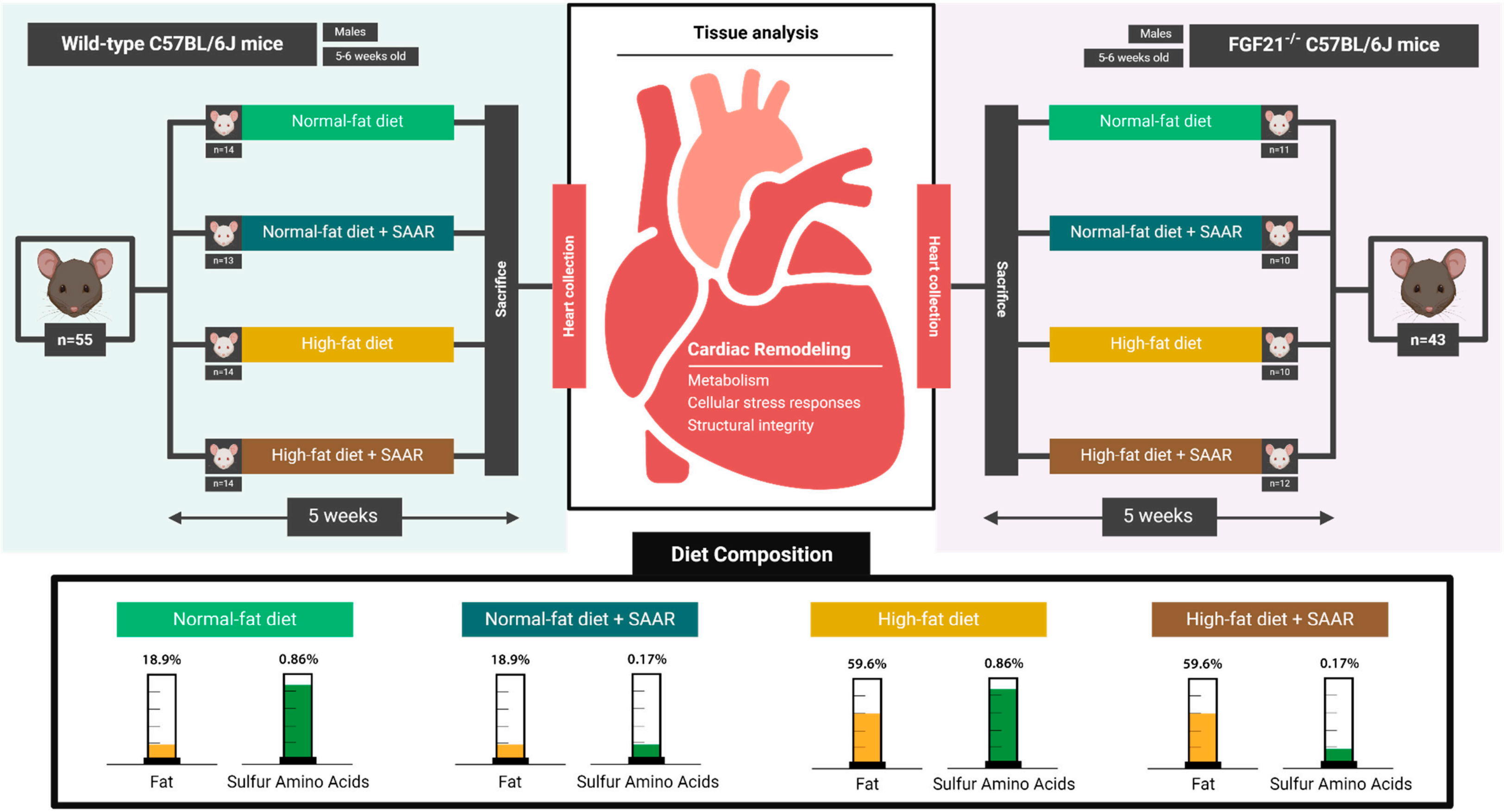

Supplementary Figure S1. Experimental design of the study. Created with Biorender.com. Abbreviations. SAAR, sulfur amino acid restriction

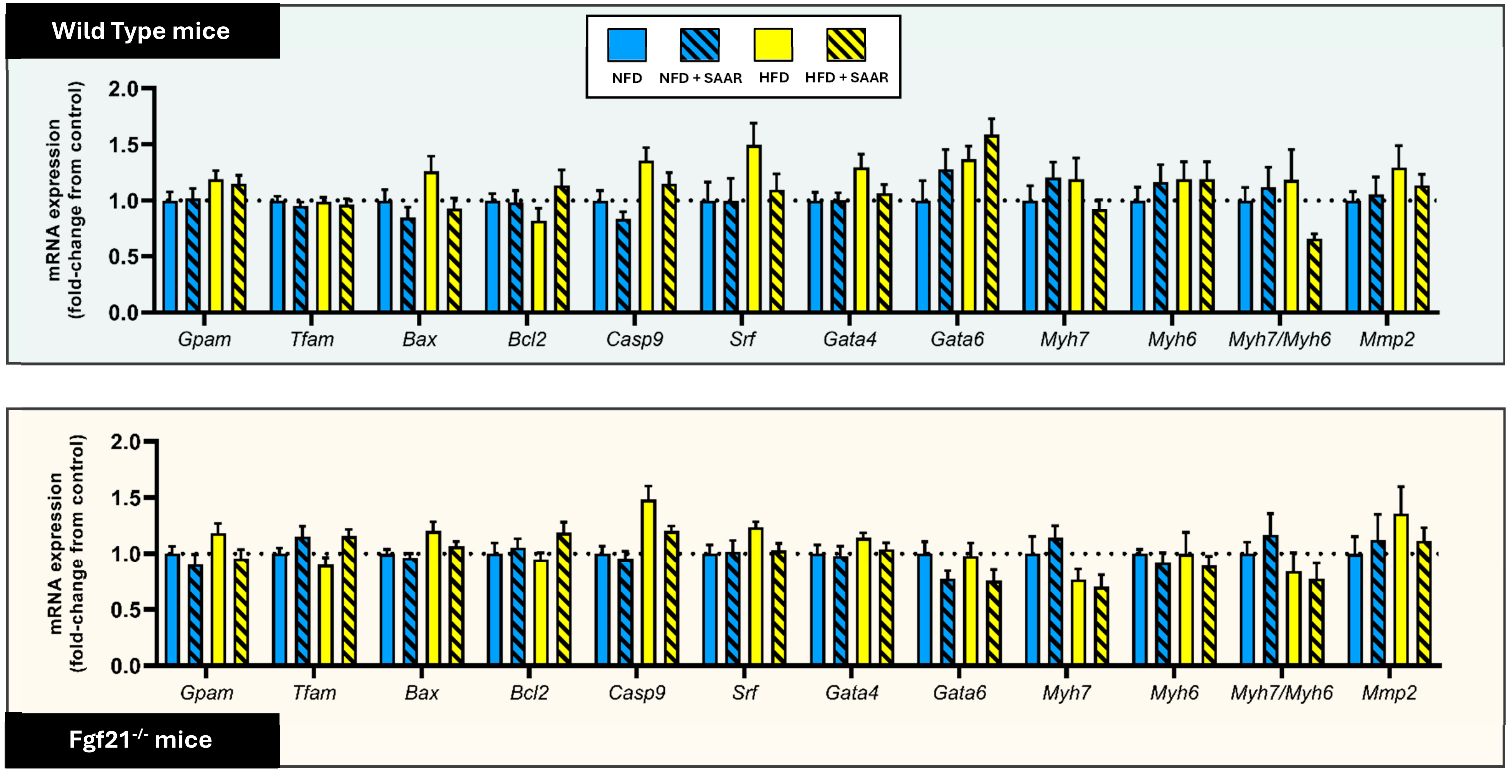

Supplementary Figure S2. Expression of other genes in the heart. mRNA expression levels were quantified via RT-PCR (n=6-11 animals per group). Statistical analysis was performed using one-way ANOVA followed by Tukey-Kramer post-hoc multiple comparison tests. Significance levels to the NFD group are denoted the * symbol: *$p<0.05$. Significance levels to the HFD group are denoted by the # symbol: #$p<0.05$.

Supplementary Table S1. Nutritional composition of each dietary regimen

Nutrient/Ingredient	Dietary group NFD (g/kg)	NFD + SAAR (g/kg)	HFD (g/kg)	HFD + SAAR (g/kg)
L-Arginine	11.2		16	
L-Lysine HCl	18.0		25.7	33
L-Histidine	3.3		4.7	
L-Isoleucine	8.2		11.7	
L-Valine	8.2		11.7	
DL-Methionine	8.6	1.72	8.6	1.72
L-Threonine	8.2		11.7	
L-Tryptophan	1.8		2.6	
L-Phenylalanine	11.6		16.6	
Glycine	23.3		33.2	
L-Leucine	11.1		11.1	
L-Glutamic Acid	27.0	33.88	46.4	45.78
**Total L-Amino Acids**	**140.5**		**200.0**	
Dextrose	200.0		50.0	
Dyetrose	50.0		143.0	
Sucrose	-		150.0	
Cornstarch	432.5		-	
Cellulose Fiber	50.0		50.0	
Corn Oil	80.0		-	
Soybean Oil w/tBHQ	-		25.0	
Lard	-		335.0	
Salt Mix #200000	35.0		35.0	
Vitamin Mix #300050	10.0		10.0	
Choline Bitartrate	2.0		2.0	
**Energy density**	**3.815 kcal/g**		**5.45 kcal/g**	
**Diet reference**	**DYETS #510072**	**DYETS #510071**	**DYETS #510244**	**DYETS #510246**

Abbreviations. NFD, normal-fat diet; NFD + SAAR, normal-fat diet with sulfur amino acid restriction; HFD, high-fat diet; HFD + SAAR, high-fat diet with sulfur amino acid restriction; tBHQ, tert-Butylhydroquinone.

Supplementary Table S2. List of targeted genes in RT-PCR

Gene		Primer sequences (5′ -> 3′)
Symbol	Name	
**Housekeeping genes**		
Gapdh	Glyceraldehyde-3-phosphate dehydrogenase	FW: ACAACTTTGGCATTGTGGAA<br>RV: GATGCAGGGATGATGTTCTG
Ppia	Peptidylprolyl isomerase A	FW: CTTCGAGCTGTTTGCAGACAAAGT<br>RV: AGATGCCAGGACCTGTATGCT
**Cardioprotective hormones**		
Fgf21	Fibroblast growth factor 21	FW: CTGGGGGTCTACCAAGCATA<br>RV: CACCCAGGATTTGAATGACC
Adipoq	Adiponectin	FW: AGGGCTCAGGATGCTACTGT<br>RV: CAGAAGTTCCCTTGGGTGGA
**Lipid utilization**		
Ppara	Peroxisome proliferator-activated receptor alpha	FW: CTGTCCGCTACTTCGAGTCC<br>RV: TGCATTGTGTGACATCCCGA
Cd36	Cluster of differentiation 36	FW: TAGTAGAACCGGGCCAGGTA<br>RV: CAGCCAGGACTGCACCAATA
Slc27a1	Solute carrier family 27 member 1	FW: TGCCACAGATCGGCGAGTTCTA<br>RV: AGTGGCTCCATCGTGTCCTCAT
Cpt1b	Carnitine palmitoyltransferase 1B	FW: CATGTATCGCCGCAAACTGG<br>RV: CCTGGGATGCGTGTAGTGTT
Acadl	Long chain acyl-CoA dehydrogenase	FW: CATTGGTGGGGACTTGCTCT<br>RV: TGGCTATGGCACCGATACAC
Lipe	Hormone-sensitive lipase	FW: GCTCATCTCCTATGACCTACGG<br>RV: TCCGTGGATGTGAACAACCAGG
Gpam	Mitochondrial glycerol-3-phosphate acyltransferase	FW: GCAAGCACTGTTACCAGCGATC<br>RV: TGCAATCAGCCTTCGTCGGAAG
**Glucose utilization**		
Slc2a1	Glucose transporter type 1	FW: GCTTCTCCAACTGGACCTCAAAC<br>RV: ACGAGGAGCACCGTGAAGATGA
Slc2a4	Glucose transporter type 4	FW: TCCTTCTATTTGCCGTCCTC<br>RV: GGTTTCACCTCCTGCTCTAA
Pfkm	Muscle phosphofructokinase	FW: CTGTTCGCTCTACCGTGAGGAT<br>RV: TTGGAACCACCTTGACCAGTCC
**Mitochondrial homeostasis**		
Ppargc1a	Peroxisome proliferator-activated receptor gamma coactivator 1-alpha	FW: ATGACCCTCCTCACACCAAACCCACAG<br>RV: CTTGAGCATGTTGCGACTGCGGTTGTG
Sirt1	Sirtuin 1	FW: CGATGACAGAACGTCACACG<br>RV: CTGCAACCTGCTCCAAGGTA
Tfam	Transcription factor A – mitochondrial	FW: GAGGCAAAGGATGATTCGGCTC<br>RV: CGAATCCTATCATCTTTAGCAAGC
Cytb	Cytochrome b	FW: GGCTACGTCCTTCCATGAGG<br>RV: TGGGATGGCTGATAGGAGGT
**Oxidative stress**		
Nfe2l2	Nuclear factor erythroid 2-related factor 2	FW: CAGCATAGAGCAGGACATGGAG<br>RV: GAACAGCGGTAGTATCAGCCAG
Catalase	Catalase	FW: TTTTCACCGACGAGATGGCA<br>RV: AAGGTGTGTGAGCCATAGCC
Gpx1	Glutathione peroxidase 1	FW: CAGTCCACCGTGTATGCCTT<br>RV: GTAAAGAGCGGGTGAGCCTT

Sod2	Superoxide Dismutase 2	FW: GCCCAAACCTATCGTGTCCA<br>RV: AGGGAACCCTAAATGCTGCC
Nox2	NADPH oxidase 2	FW: GAGGTTGGTTCGGTTTTGGC<br>RV: TGCACAGCAAAGTGATTGGC
**Inflammation**		
Tnf	Tumor necrosis factor alpha	FW: GGTGCCTATGTCTCAGCCTCTT<br>RV: GCCATAGAACTGATGAGAGGGAG
Il1b	Interleukin 1 beta	FW: TGCCACCTTTTGACAGTGATG<br>RV: TTCTTGTGACCCTGAGCGAC
Ifng	Interferon gamma	FW: GAGGTCAACAACCCACAGGT<br>RV: GGGACAATCTCTTCCCCACC
Ccl2	C-C motif chemokine ligand 2	FW: GCTACAAGAGGATCACCAGCAG<br>RV: GTCTGGACCCATTCCTTCTTGG
**Apoptosis**		
Bax	BCL2-associated X protein	FW: AGGATGCGTCCACCAAGAAG<br>RV: CTTGGATCCAGACAAGCAGC
Bcl2	B-cell lymphoma 2	FW: CCTGTGGATGACTGAGTACCTG<br>RV: AGCCAGGAGAAATCAAACAGAGG
Casp3	Caspase 3	FW: GAGCTTGGAACGGTACGCTA<br>RV: CCGTACCAGAGCGAGATGAC
Casp9	Caspase 9	FW: GCTGTGTCAAGTTTGCCTACCC<br>RV: CCAGAATGCCATCCAAGGTCTC
**Pathological hypertrophy**		
Srf	Serum response factor	FW: CCGCGTGAAGATCAAGATGGAGTT<br>RV: TGCCAGGTAGTTGGTGATGGGGAA
Gata4	GATA binding protein 4	FW: ACCAAAGCCTGCCTATGGCC<br>RV: CCGCCAGGGACCCAGTAGTC
Gata6	GATA binding protein 6	FW: TGAACGGGACGTACCACCACCACC<br>RV: ACAGTTCACGCACTCGCGGCTCTC
Nfatc2	Nuclear factor of activated T cells, cytoplasmic, calcineurin-dependent 2	FW: ACTTCACAGCGGAGTCCAAGGT<br>RV: GGATGTGCTTGTTCCGATACTCG
Nppa	Natriuretic peptide A	FW: CTTCTCCATCACCAAGGGCTT<br>RV: GGATTTGCTCCAATATGGCCT
Nppb	Natriuretic peptide B	FW: TTTGGGCTGTAACGCACTGA<br>RV: CACTTCAAAGGTGGTCCCAGA
Myh6	Myosin heavy chain 6	FW: ATAAAGGGGCTGGAGCACTG<br>RV: TCATTCTGTCACTCAAACTCTGGT
Myh7	Myosin heavy chain 7	FW: AGGGCGACCTCAACGAGAT<br>RV: CAGCAGACTCTGGAGGCTCTT
**Fibrosis**		
Col1a1	Collagen, type I, alpha 1	FW: GACTGGAAGAGCGGAGAGTACTG<br>RV: CCTTGATGGCGTCCAGGTT
Col3a1	Collagen, type III, alpha 1	FW: AGTGGCCATAATGGGGAACG<br>RV: CACCTTTGTCACCTCGTGGA
Mmp2	Matrix metallopeptidase 2	FW: CAAGGATGGACTCCTGGCACAT<br>RV: TACTCGCCATCAGCGTTCCCAT
Mmp9	Matrix metallopeptidase 9	FW: GCTGACTACGATAAGGACGGCA<br>RV: TAGTGGTGCAGGCAGAGTAGGA
